# Draft Genome Sequence of *Leifsonia xyli* subsp. *xyli* Strain gdw1

**DOI:** 10.1128/genomeA.01128-16

**Published:** 2016-10-20

**Authors:** Jihua Wang, Li Wang, Gan Cao, Muqing Zhang, Ying Guo

**Affiliations:** a Crops Research Institute, Guangdong Academy of Agricultural Sciences, Guangzhou, China; b Guangdong Key Laboratory for Crops Genetic Improvement, Guangzhou, China; c State Key Laboratory for Subtropical Agri-Bioresources Conservation and Utilization, Guangxi University, Nanning, China; d Fujian Institute of Subtropical Botany, Xiamen, Fujian, China

## Abstract

Here, we report the draft genome sequence of *Leifsonia xyli* subsp. *xyli* strain gdw1, isolated from the stem of Badila sugarcane located at the Guangdong Key Laboratory for Crops Genetic Improvement (Guanzhou, China), that causes ratoon stunting disease of sugarcane. The *de novo* genome of *Leifsonia xyli* subsp. *xyli* was assembled with 48 scaffolds and a G+C content of 67.68%, and contained 2.6 Mb bp and 2,838 coding sequences.

## GENOME ANNOUNCEMENT

The Gram-positive and nutritionally fastidious bacterium *Leifsonia xyli* subsp. *xyli* (Lxx), the causal agent of ratoon stunting disease of sugarcane (RSD), is a major contributor to substantial sugarcane biomass losses worldwide. It impairs the development of the ratoon or stubble plants by shortening the internodes (1). Because Lxx is difficult to culture *in vitro*, the use of PCR is conventional. Sequencing the genome of Lxx will allow for the exploitation of a specific DNA probe to detect its presence in sugarcane (2). To date, two genomes of *Leifsonia xyli* subsp. *xyli* have been sequenced, however, only the sequence of strain CTCB07 is published (3).

*Leifsonia xyli* subsp. *xyli* strain gdw1 was isolated from the stem of sugarcane located at the Guangdong Key Laboratory for Crops Genetic Improvement (Guanzhou, China) and identified with 16S rRNA and Lxx sequencing (4). Genomic DNA of strain gdw1 was extracted from bacterial pure cultures by the CTAB protocol (5) and sent for sequencing to Aihua Technologies, Ltd. (Nanjing, China). A single 300-bp library was prepared by Covaris M220 with the TruSeq DNA sample prep kit and TruSeq PE cluster kit. The total nucleotide of 2,076,594,300 bp was provided by Illumina Hiseq sequencing with the Truseq SBS kit, and was approximately 770× coverage of the entire gdw1 strain with a genome size of approximately 2.60 Mb. The cleaned reads were assembled by SOAPdenovo v2.04 and GapCloser v1.12 to get the final draft genome that includes 48 scaffolds of 2,548,085 bp, with a total G+C content of 67.68%, bases in large scaffolds of 2,543,572 bp, largest length of 312,666 bp, *N*_50_ of 101,891 bp, and *N*_90_ of 28,358 bp (6). The gene prediction was carried out using Glimmer 3.02 (http://ccb.jhu.edu/software/glimmer/index.shtml). A total of 2,838 coding sequences were predicted and annotated in total base pairs of 2,154,303 bp and a mean length of 759 bp. Specifically, of the 51 RNA regions, three encoding rRNA and 48 encoding tRNA were identified by Barrnap 0.4.2 (http://www.vicbioinformatics.com/software.barrnap.shtml) and tRNAscan-SE v1.3.1 (http://lowelab.ucsc.edu/tRNAscan-SE/). One antibiotic-resistant gene and 279 virulence factors were identified using BLAST against the NCBI database (7).

From the annotated sequences of *Leifsonia xyli* subsp. *xyli* strain gdw1, five genomes of *xyli* have been compared. The draft genome sequence of *Leifsonia xyli* subsp. *xyli* strain gdw1 may help to design special primers for early detection of RSD (8).

### Accession number(s).

This whole-genome shotgun project of *Leifsonia xyli* subsp. *xyli* strain gdw1 has been deposited at DDBJ/EMBL/GenBank under the accession no. LNZG00000000. The version described in this paper is version LNZG01000000.
